# Essential Oils from Citrus Peels Promote Calcium Overload-Induced Calcicoptosis in U251 Cells

**DOI:** 10.3390/antiox14010011

**Published:** 2024-12-25

**Authors:** Yurong Li, Juanjuan Wei, Zimao Ye, Chen Ji, Wenji Li, Li Xu, Zhiqin Zhou

**Affiliations:** 1 Key Laboratory of Agricultural Biosafety and Green Production of Upper Yangtze River (Ministry of Education), College of Horticulture and Landscape Architecture, Southwest University, Beibei District, Chongqing 400715, China; yellor326815@163.com (Y.L.); wei000319@email.swu.edu.cn (J.W.); yezimao@email.swu.edu.cn (Z.Y.); 1834606465@163.com (C.J.); 2 School of Design, Chongqing Industry Polytechnic College, Chongqing 401120, China; liwj@cqipc.edu.cn; 3 College of Sericulture, Textile and Biomass Sciences, Southwest University, Beibei District, Chongqing 400715, China; 4 Southwest Institute of Fruits Nutrition, Banan District, Chongqing 400054, China

**Keywords:** citrus, essential oil, glioblastoma, calcium overload, calcicoptosis

## Abstract

Citrus peel essential oils (CPEOs) have demonstrated substantial medicinal potential for glioblastoma treatment because of their extensive antitumor effects, low potential for drug resistance, and ability to cross the human blood–brain barrier. In this study, the chemical compositions of five CPEOs were analyzed via gas chromatography–mass spectrometry (GC-MS). CCK8 assays were used to evaluate the ability of five CPEOs to inhibit U251 human glioblastoma cells, and XLB and RA were selected for further investigation. Through wound healing assays and cell cycle and apoptosis analyses via flow cytometry, it was revealed that these CPEOs inhibited cell migration, arrested the cell cycle at G1/G0, and induced apoptosis with similar levels of inhibition. After CPEOs treatment, the intracellular Ca^2+^ content and reactive oxygen species levels in U251 cells increased significantly, whereas the mitochondrial membrane potential decreased. Additionally, the antioxidant enzyme system (SOD, POD, CAT, and GR) and the nonenzymatic defense system (GSH) were inhibited, leading to an increase in lipid peroxidation. qRT–PCR indicated the significant upregulation of intracellular calcium ion signaling pathways and the upregulation of mitochondrial apoptosis-related genes. Additionally, the activation of calcicoptosis-related indicators induced by the CPEOs could be reversed by inhibitor treatment, confirming that both of the selected CPEOs inhibit tumors by activating calcicoptosis-related pathways. These findings highlight the immense potential of CPEOs in healthcare and pharmaceutical applications by not only providing a scientific basis for the potential application of CPEOs in the treatment of glioblastoma but also offering new insights for the development of novel antitumor drugs.

## 1. Introduction

Primary brain tumors account for approximately 2% of all tumors, with gliomas being the most common among them. Depending on the age of the patient, 40% to 90% of gliomas are malignant [1]. Glioblastoma multiforme (GBM) is one of the most aggressive and poorly prognosed types of glioma. Among all glioma types, GBM has a high incidence, high mortality, and an extremely poor prognosis [2]. Research has shown that the average survival time for patients with GBM is only 6–14 months, thus posing a serious threat to the lives and health of patients [3,4]. Despite significant efforts to develop new therapies, there has been no breakthrough in GBM treatment since the standard treatment regimen proposed by Stupp et al. [4], which involves surgical resection followed by radiotherapy and chemotherapy with temozolomide (TMZ), was introduced. For decades, the presence of the blood–brain barrier (BBB) has posed a significant challenge, preventing many antitumor drugs from entering the brain [2]. Even with the combination of cutting-edge medical treatments such as electric field therapy, immunotherapy, and targeted drugs [5], in most cases, the tumor will inevitably recur because of its rapid growth rate and the development of target resistance [4,6].

The discovery of natural plant-based products is considered a milestone in the history of healthcare, and their introduction to the market in combination with synthetic drugs has addressed numerous health issues [7]. Two-thirds of the drugs currently used to treat cancer are either naturally sourced or derived from natural products, and the US Food and Drug Administration (FDA) approved 547 natural products and their derivatives for use as medications from 1827 to 2013 [8]. Natural products with antitumor properties include terpenes, alkaloids, flavonoids, quinones, saponins, and various primary and secondary metabolites [9,10,11]. The peels of citrus fruits are rich in essential oils (CPEOs), which are primarily present in the extremely small secretory glands [12]. CPEOs are widely used because of their observed natural properties, such as antitumor, antioxidant, anti-inflammatory, and antibacterial effects, which gives them versatile application prospects, primarily in the health, agriculture, cosmetics, and biomedical pharmaceutical industries [13,14,15]. CPEOs have demonstrated promising anticancer potential in recent research [16] by inhibiting various types of tumor cells. Additionally, as natural products, CPEOs are classified and generally recognized as safe (GRAS) by the FDA [17].

Calcicoptosis, a novel form of programmed cell death induced by intracellular Ca^2+^ overload, leads to mitochondrial dysfunction and excessive reactive oxygen species (ROS) production; thus, targeting calcicoptosis is a highly promising anticancer mechanism [18,19]. Mitochondria are important organelles for ROS production [20], but when they are damaged, mitochondria convert some of the residual oxygen into superoxide [21]. When the rate of intracellular ROS production exceeds the antioxidant capacity, ROS accumulation increases, leading to cell damage and the occurrence of oxidative stress [22]. There is a link between oxidative stress and Ca^2+^ overload. Under oxidative stress, the ability of a cell to regulate Ca^2+^ gradually decreases, leading to the continuous accumulation of Ca^2+^ and calcium overload, which further damages the mitochondria [23]. Studies have demonstrated that certain CPEOs components, such as limonene, β-caryophyllene, and α-pinene, can induce mitochondrial dysfunction and stimulate the production of ROS in cancer cells without increasing the oxidative stress in normal cells [24,25]. The unique lipophilic nature of CPEOs enhances their cellular absorption, endowing them with good bioavailability in systemic circulation. Furthermore, studies have shown that CPEOs can enter the bloodstream and cross the BBB [26,27]. Because CPEOs can act on multiple targets simultaneously, developing resistance to these agents is difficult [28], which demonstrates their great potential in the fight against glioma. However, there is currently limited research on the mechanisms by which CPEOs induce Ca^2+^ accumulation and overload in cancer cells and cause calcicoptosis.

In this study, five kinds of citrus peels were selected as the raw materials. After the essential oils (EOs) were extracted from the peels, their compositions and contents were analyzed via gas chromatography–mass spectrometry (GC–MS). Two citrus varieties with significant antiproliferative activity were identified through cell activity screening. Furthermore, the human glioblastoma cell line U251 was used in this study as the culture model to focus on the phenomenon of calcicoptosis induced by CPEOs and explore their inhibitory effects. The aim of this study was to evaluate the potential of applying CPEOs from different citrus varieties to combat glioma and to elucidate the specific mechanisms by which they exert their antiglioma effects through calcicoptosis-related pathways.

## 2. Materials and Methods

### 2.1. Plant Material

Five different cultivated citrus materials were selected for this study, as detailed in Table 1. The five types of citrus fruits were all harvested from local orchards in December 2022. For each cultivar, plants with uniform growth vigor, healthy tree structures, and free from obvious pests and diseases, as well as other abiotic stresses, were selected for sampling. The fruits were consistent in terms of their shape, size, and color, had no visual defects or damage, and were immediately delivered to the laboratory of Southwest University (Chongqing, China) after harvest. The citrus materials were soaked in ultrapure water to remove the surface dust and impurities, air dried at 25 °C, and stored at 4 °C for future use.

### 2.2. Extraction of CPEOs

In accordance with the methods of Yao et al. [29], for this study, CPEOs were extracted from five different citrus varieties via steam distillation. First, 200 g of the cleaned, crushed peel was added to a round-bottom flask. Then, 12 g of sodium chloride and 800 g of deionized water were added for the purpose of steam distillation. The temperature of the electric heating jacket (DRT-SX, GreatWall, Zhengzhou, China) was set to 100 °C, and the mixture underwent steam distillation for 4 h. The distillate was collected in a glass receiver where the essential oil was allowed to float above the hydrosol. After the distillation process, the hydrosol was drained and the essential oil was collected. After filtration, the CPEOs were dried over 0.3% anhydrous sodium sulfate. The collected CPEOs were then sealed in brown bottles and stored at −80 °C for future use.

### 2.3. GC-MS

The volatile components of the five EOs were analyzed with reference to the method described by Yao et al. [29]. A Shimadzu 2010 Ultra GC–MS (Shimadzu, Kyoto, Japan) equipped with an Rxi-5ms capillary gas chromatography column (30 m × 0.25 mm × 0.25 μm) was used. The inlet temperature was set at 250 °C, and the transfer line temperature was also set at 250 °C. Helium, with a purity of >99.99% and a flow rate of 1 µL/min, was used as the carrier gas with a split ratio of 10:1. One microliter of the citrus EO to be tested was injected. Under MS conditions, the ion source temperature was set at 200 °C, and ionization was performed by electron impact (EI) with an electron energy of 70 eV and a scan range of 35–500 *m*/*z*. Finally, qualitative and quantitative analyses were conducted. Substances with a similarity of 80% or above were screened out and matched with computer libraries (NIST 2008 and Flavor 2.0) via the detected mass spectra of the compounds. The relative content was calculated via the peak area normalization method, with three repetitions for each sample.

### 2.4. Cell Culture and Reagents

U251 human glioblastoma cells were purchased from Procell Life Science & Technology Co., Ltd. (Wuhan, China). The U251 cells were cultured in Dulbecco’s modified Eagle’s medium (DMEM) (Procell, Wuhan, China) supplemented with 10% fetal bovine serum (FBS) (BI, Montevideo, Uruguay, South America), 1% penicillin–streptomycin, and 0.01 mg/mL insulin. The cells were cultured in a constant-temperature incubator at 37 °C with 5% CO_2_ and incubated for 24 h before the treatment with the extracts.

### 2.5. Cell Viability Assays

U251 cells were cultured to the exponential growth phase and seeded into 96-well plates at a density of 1 × 10^4^ cells per well. After 24 h of incubation, the culture medium was replaced with fresh medium containing different concentrations of the CPEOs (ranging from 45 to 120 µg/mL) for the treatment. Fresh medium containing 0.5% dimethyl sulfoxide (DMSO) was used as the control medium (CK). After 24 h of CPEO treatment, the culture medium was discarded, and the cells were washed with PBS. A CCK8 kit (Biosharp, Hefei, China) was subsequently used to evaluate the cell viability, and the absorbance values of the CK and different treatment groups were measured at 450 nm. We utilized the software OriginPro 2021 to calculate the percent cell viability in the control and different treatment groups and perform curve fitting to determine the half-maximal inhibitory concentration (IC_50_) value. The experiment was independently repeated three times to ensure the reliability and reproducibility of the results.

### 2.6. Cell Proliferation Assays

The antiproliferative effects of the CPEOs were analyzed via the BeyoClick^TM^ EdU Cell Proliferation Kit (Alexa Fluor 555, Beyotime, Shanghai, China). The experiment was conducted according to the manufacturer’s instructions. After 24 h of CPEO treatment, the cells were incubated with 10 µM 5-ethynyl-2′-deoxyuridine (EDU) solution at 37 °C for 2 h and then fixed with 4% paraformaldehyde (Beyotime, Shanghai, China) for 15 min. After the cells were washed three times with PBS, they were incubated with Hoechst 33342 for 10 min. Finally, the cells were analyzed and imaged via a fluorescence microscope (Olympus U-HGLGPS, Shanghai, China). The experiment was independently repeated three times to ensure the reliability and reproducibility of the results.

### 2.7. Wound Healing Assays

U251 cells (1 × 10^6^ cells/well) were seeded in 6-well plates and cultured for 24 h. A cross-scratch was made on the cell monolayer with a sterile 200 μL pipette tip, after which the cells were washed with PBS to remove any loosely attached cells or debris. Then, the CPEOs were added, and their effect on wound healing was monitored. The cells were photographed at 0 and 24 h using an inverted fluorescence microscope (Olympus U-HGLGPS, Suzhou, China). The experiment was independently repeated three times. The migration rate was analyzed with ImageJ software (version 1.4.3.67).

### 2.8. Cell Cycle and Apoptosis Analyses

The cell cycle and apoptosis were analyzed via a Cell Cycle Analysis Kit (propidium iodide (PI)) (Biosharp, Hefei, China) and an Annexin V-FITC/PI Apoptosis Kit (Elabscience, Wuhan, China), respectively. After the cells were treated with the CPEOs for 24 h, they were digested with trypsin and centrifuged at 1000× *g* for 5 min. The supernatant was discarded, and the cells were washed twice with PBS at 4 °C. Then, 70% ethanol prechilled at 4 °C was added, and the cells were fixed at 4 °C for 12 h. After washing once with PBS at 4 °C, 0.5 mL of PI was added, and the cells were stained at 37 °C for 30 min for cell cycle analysis. Then, the cells were collected, washed twice with PBS at 4 °C, and resuspended in 1× Annexin V buffer. Subsequently, 2.5 μL of V-FITC and 2.5 μL of PI were added, and the cells were stained in the dark for 15 min for apoptosis analysis. The cell cycle distribution and degree of apoptosis were determined via flow cytometry (BD, PerkinElmer, Shelton, CT, USA). A total of 2 × 10^4^ cells from each sample were counted. The experiments were independently repeated three times.

### 2.9. Detection of the Intracellular Calcium Ion Content

To detect the intracellular calcium ion content, the cells were stained with the Fluo-4 Calcium Assay Kit (Beyotime, Shanghai, China) and DAPI staining solution (Beyotime, Shanghai, China). After the treatment with the CPEOs for 24 h, the culture medium was removed, and the cells were washed twice with PBS. Then, 1 mL of Fluo-4 staining solution was added, and the cells were incubated in the dark at 37 °C for 30 min. The staining solution was aspirated, and the cells were washed once with PBS. Next, 1 mL of DAPI staining solution was added, and the cells were incubated in the dark at 37 °C for 5 min. The DAPI staining solution was aspirated, and the cells were washed twice with PBS. The fluorescence intensity was measured via a multimode microplate reader at an emission wavelength of 490 nm. Finally, images were captured via a fluorescence microscope (Olympus U-HGLGPS, Shanghai, China). The experiments were independently repeated three times.

### 2.10. Inhibitors of Calcicoptosis

The cytotoxicity of the calcicoptosis inhibitors A23187 and nifedipine (NIP), obtained from MedChemExpress (New Jersey, USA), was evaluated via CCK8 assays. The addition of A23187 and NIP (10 μM) largely enhances the cell viability treated by CPEOs (Figure S1). Subsequently, U251 cells were pretreated with each of the two calcicoptosis inhibitors for 1.5 h. After the culture medium was aspirated, the cells were treated with the CPEOs for 24 h. Next, the mitochondrial membrane potential (MMP), ROS levels, related enzyme activities, and glutathione (GSH) and malondialdehyde (MDA) levels were measured, and a quantitative reverse transcription polymerase chain reaction (qRT–PCR) analysis was performed.

### 2.11. Analysis of the MMP

The Enhanced Mitochondrial Membrane Potential Assay Kit with JC-1 was purchased from Solarbio Life Science and Technology Ltd. (Beijing, China). After the cells were treated with the CPEOs for 24 h, the culture medium was aspirated, and the cells were incubated with JC-1 working solution in the dark at 37 °C for 20 min. After incubation, the cells were washed twice with JC-1 buffer solution. Next, the fluorescence intensity was measured via a multimode microplate reader at an excitation wavelength of 490 nm and an emission wavelength of 530 nm. Images of the treated cells were captured via an inverted fluorescence microscope. The experiments were independently repeated three times.

### 2.12. Measurement of the ROS Levels

The fluorescent probe DCFH-DA (10 mM) (Biosharp, Hefei, China) (also available from Solarbio, Beijing, China) was used to evaluate the intracellular ROS levels. After the cells were treated with the CPEOs for 24 h, the culture medium was aspirated, and the cells were washed twice with PBS. DCFH-DA (diluted to 10 μM in 1 mL of serum-free culture medium) was subsequently added. After incubation in the dark at 37 °C for 30 min, the cells were washed three times with serum-free medium to thoroughly remove any residual DCFH-DA that had not penetrated the cells. The fluorescence intensity of the cells was then measured via a fluorescence microscope (Olympus U-HGLGPS, Beijing, China). The experiment was independently repeated three times.

### 2.13. Oxidative Factor Detection

The enzyme activity assay kits, content assay kits, and BCA protein assay kit were purchased from Solarbio Life Science and Technology Ltd. (Beijing, China). The cells were treated with the CPEOs for 24 h, after which the culture medium was aspirated, and the cells were digested with trypsin. The cells were then collected and added to extraction solution for the enzyme activity determination and oxidative stress marker content measurements. The activities of superoxide dismutase (SOD) and peroxidase (POD) were measured according to the manufacturer’s instructions, and the absorbance values were measured at 450 nm and 470 nm via a microplate reader. The activities of glutathione reductase (GR) and catalase (CAT) were determined via ultraviolet colorimetry, and the absorbance values were measured at 340 nm and 240 nm, respectively, via a microplate reader. The levels of MDA and GSH were detected using their respective assay kits, and the absorbance values were measured via a microplate reader. The protein content was measured with a BCA assay kit. Each experiment was independently repeated three times.

### 2.14. qRT–PCR

A Total RNA Extraction Kit was purchased from TIANGEN (Beijing, China), while the Reverse Transcription Kit and SYBR Green Master Mix were obtained from Vazyme (Nanjing, China). RNA was extracted from the cells following the manufacturer’s instructions. The isolated total RNA was subsequently reverse transcribed into cDNA according to the protocol provided. The qRT–PCR analysis was performed on a Bio-Rad CFX96 system using gene-specific primers. To quantify the PCR products, SYBR Green Master Mix was used according to the manufacturer’s instructions. All the data were normalized to the level of GAPDH. The experiment was independently repeated three times. The primer sequences are provided in Supplementary Table S1.

### 2.15. Statistical Analysis

Each experiment was repeated three times, and the results are presented as the means ± standard deviations (SDs). Graphs were plotted with OriginPro 2021 software, and the statistical significance between multiple samples was determined via an analysis of variance (ANOVA) and Duncan’s test, which were performed using SPSS 20.0 software, and the significance level was set at *p* < 0.05 to assess the statistical significance.

## 3. Results

### 3.1. Analysis of the Composition of Five Types of CPEOs

**Figure 1:**
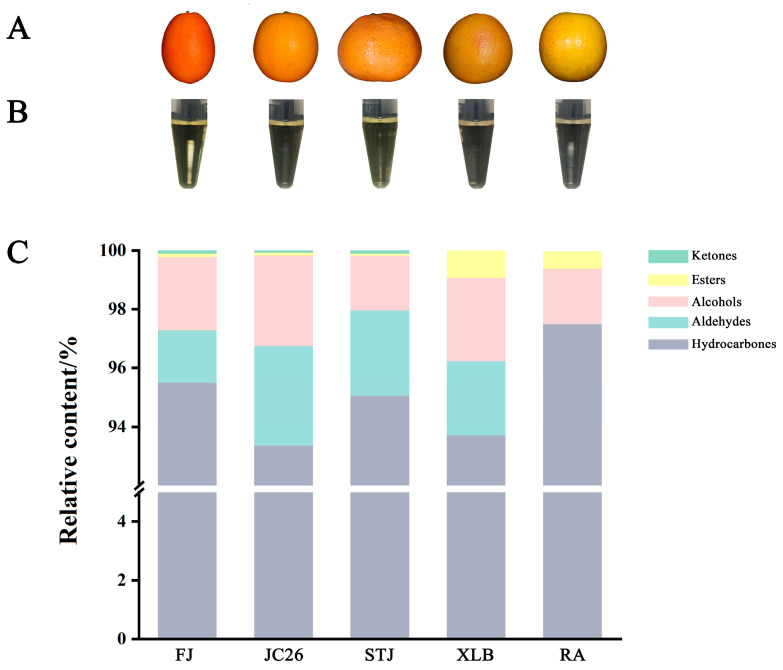
The relative content and composition of five CPEOs. (**A**) Phenotype of five citrus fruits. (**B**) The colors of the five CPEOs. (**C**) The differences in the relative content and composition of five EOs components. FJ: FengJieQiCheng peel essential oil; JC26: JinCheng26 peel essential oil; STJ: ShaTangJu peel essential oil; XLB: XingLuBiXiYou peel essential oil; and RA: RongAnJinDan fruit peel essential oil.

As shown in Figure 1, the essential oils (EOs) of FJ, JC26, and STJ contain chemical components such as hydrocarbons, aldehydes, ketones, alcohols, and esters. However, ketones were not detected in the EOs of XLB, and neither aldehydes nor ketones were detected in the EOs of RA. As shown in Figure S2 and Table S2, a total of 46, 46, 44, 49, and 51 volatile components were detected in the EOs of the five varieties, respectively. In the EOs of FJ, JC26, STJ, and XLB, the volatile components with relatively high content include D-limonene, β-myrcene, α-pinene, linalool, and octanal. Except for octanal, which was not detected in the RA EO sample, the other four components are consistent with those found in the other EOs. Among the five EOs, D-limonene has the highest relative content, accounting for 81.88 ± 0.02%, 83.24 ± 0.6%, 85.03 ± 0.65%, 82.37 ± 0.52%, and 85.24 ± 0.26% of the detected aroma components, respectively. RA EOs have the highest relative content of hydrocarbon compounds among the five EOs, at 97.5%, while JC26 EOs have the lowest, at 93.36%. The highest relative content of aldehydes is found in JC26 EOs, at 3.4%. The highest relative content of alcohols is also found in JC26 EOs, while the lowest is in STJ EOs. XLB EOs have the greatest variety of esters, with a total of five types.

### 3.2. Effects of the CPEOs on U251 Cell Viability

**Figure 2:**
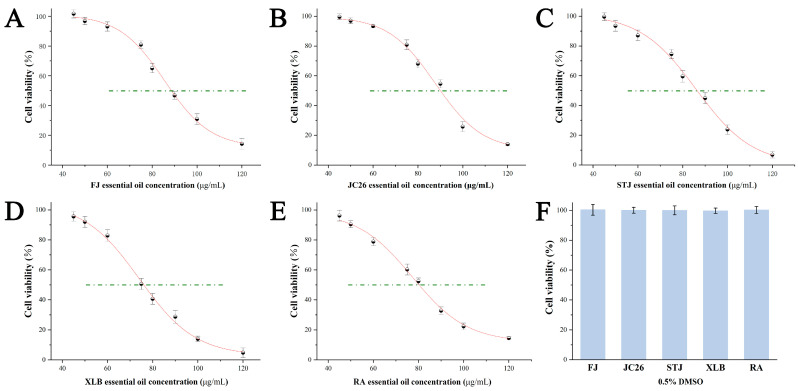
Effect of five CPEOs on the growth of U251 cells. (**A**–**E**) U251 cells were treated with various concentrations of FJ, JC26, STJ, XLB, and RA EOs for 24 h. (**F**) The control was treated with 0.5% DMSO. The data represent the mean ± SD. The green dashed line indicated a cell viability rate of 50%.

To investigate the effects of the CPEOs on U251 cell growth, we used CCK8 assays to evaluate the U251 cell viability after 24 h of incubation with different concentrations (120, 100, 90, 80, 75, 60, 50, or 45 μg/mL) of various EOs (Figure 2A–E). The control group was treated with 0.5% DMSO, which did not affect the cell viability (Figure 2F). The U251 cell viability decreased significantly in a dose-dependent manner upon EO treatment. The IC_50_ values for the five EOs (FJ, JC26, STJ, XLB, and RA) were 88.63 ± 1.56, 90.23 ± 1.49, 86.02 ± 1.67, 76.59 ± 1.69, and 80.05 ± 2.60 μg/mL, respectively. The lower IC_50_ values for the EOs XLB and RA indicate that they have stronger inhibitory effects on cells at lower concentrations. Therefore, in subsequent experiments, the EOs XLB and RA at concentrations of 40 and 80 μg/mL (denoted XLB40 and XLB80; and RA40 and RA80, respectively) were used for treatment, and the 0.5% DMSO-treated group was denoted CK.

### 3.3. Effects of the CPEOs on U251 Cell Proliferation and Migration

**Figure 3:**
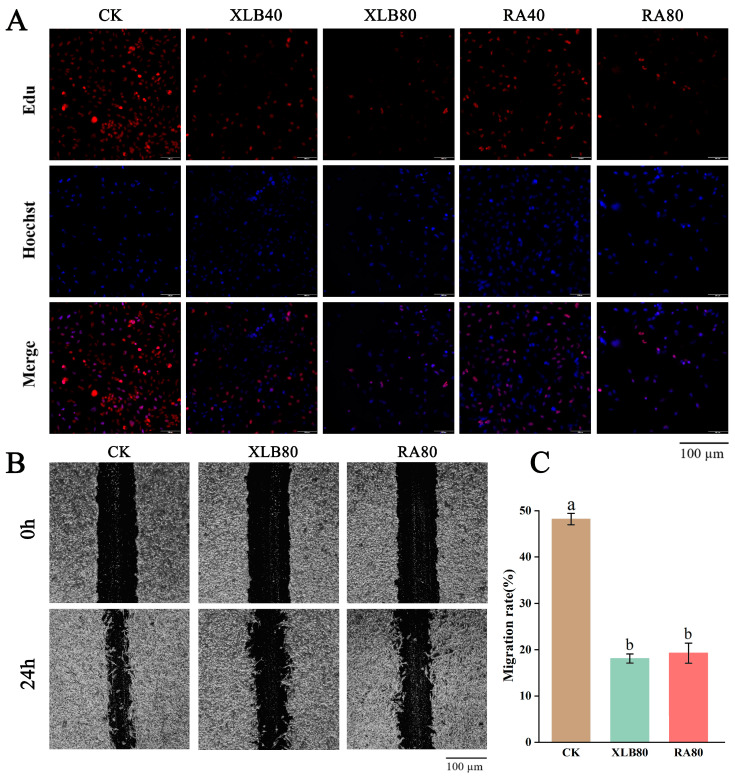
Effect of the CPEOs on proliferation and migration in U251 cells. (**A**) XLB and RA EOs can inhibit cell proliferation. Proliferating cells and cell nuclei were stained with BeyoClick™ EdU-555 kit and Hoechst 33342. (**B**) The cell migration ability of U251 cells was determined using the wound healing assay, where the cells were treated with XLB and RA EOs in serum-free medium for 24 h. (**C**) Statistical data of migration rate (%). The concentrations of XLB40 and RA40 were 40 µg/mL, while those of XLB80 and RA80 were 80 µg/mL. CK was treated with 0.5% DMSO. The data represent the mean ± SD. Values followed by different superscripts (a,b) are significantly different (*p* < 0.05).

To investigate the effects of the two CPEOs on the U251 cell proliferation, we performed EdU staining assays. As shown in Figure 3A, we observed a significant decrease in red fluorescence after treatment with XLB40 and RA40 compared with that after CK treatment, indicating that XLB40 and RA40 inhibited the U251 cell proliferation. As the concentrations of XLB and RA increased, the DNA synthesis in the cells was significantly and dose-dependently inhibited.

In this study, we also investigated the U251 cell migration after the CPEO treatment via cell wound healing assays (Figure 3B). The results (Figure 3C) revealed that, compared with CK, XLB80 and RA80 significantly inhibited cell wound healing by 30.11% and 28.95%, respectively.

### 3.4. Effects of the CPEOs on the U251 Cells Cycle

**Figure 4:**
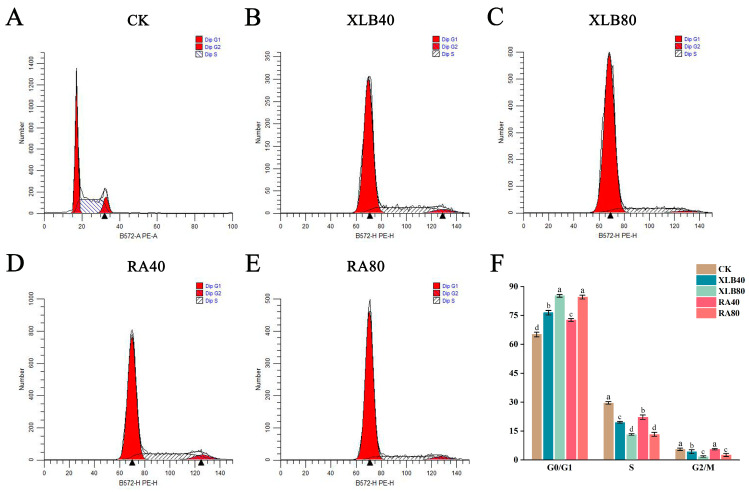
Effects of the CPEOs on the U251 cells cycle. (**A**–**E**) Determination of cell cycle distribution by flow cytometry. (**F**) The proportions of cell populations in G0/G1, S, and G2/M phases after treatment with XLB and RA EOs. The concentrations of XLB40 and RA40 were 40 µg/mL, while those of XLB80 and RA80 were 80 µg/mL. CK was treated with 0.5% DMSO. The data are presented as mean ± SD. Values followed by different superscripts (a–d) are significantly different (*p* < 0.05).

The effects of the CPEOs on the cell cycle were investigated via PI staining experiments (Figure 4). The results revealed that after 24 h of treatment with XLB and RA, the number of cells in the S phase decreased significantly, whereas the number of cells in the G1/G0 phase increased significantly, with the most pronounced effect observed at 80 µg/mL. Specifically, the proportions of cells in the S phase decreased by 10.03% and 16.32% in the XLB40 and XLB80 groups, respectively, whereas the proportions of cells in the G1/G0 phase increased from 65.07% ± 1.18% to 76.40% ± 1.09% and 85.13% ± 0.66%, respectively. Compared with that in the CK group, in the RA40 and RA80 groups, the proportions of cells in the S phase decreased by 7.42% and 16.34%, respectively, while the proportions of cells in the G1/G0 phase increased by 7.41% and 19.32%, respectively. These findings indicate that the CPEOs dose-dependently impeded the transition of U251 cells from the G1/G0 phase to the S phase, inducing G1/G0 arrest and reducing the number of cells in the S phase (Figure 4F).

### 3.5. Effects of the CPEOs on U251 Cell Apoptosis

**Figure 5:**
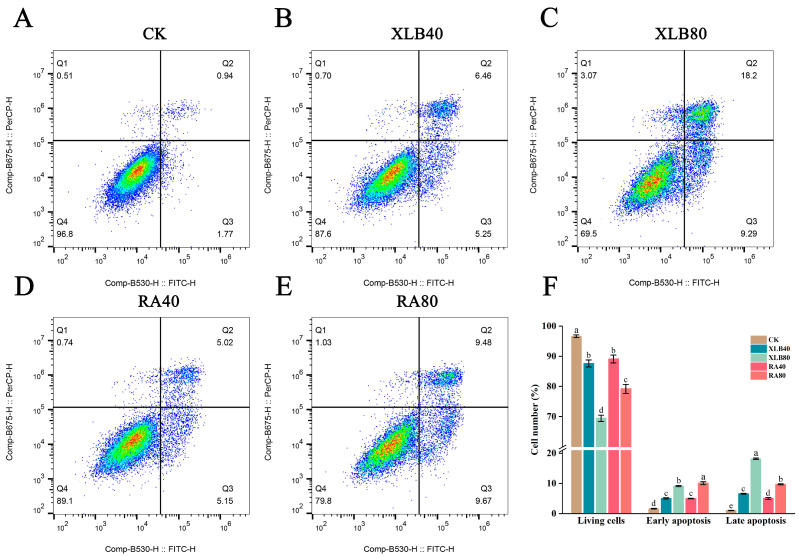
Effects of the CPEOs on U251 cell apoptosis. (**A**–**E**) Apoptotic cells were assessed by flow cytometry after Annexin V-FITC and PI staining. Q1 represents necrotic cells; Q2 represents late apoptotic cells; Q3 represents early apoptotic cells; Q4 represents viable cells. (**F**) Living cells, early and late apoptotic cell rates. The concentration of XLB40 and RA40 was 40 µg/mL, while that of XLB80 and RA80 was 80 µg/mL. CK was treated with 0.5% DMSO. The data are presented as the mean ± SD. Values followed by different superscripts (a–e) are significantly different (*p* < 0.05).

Figure 5 shows the percentages of viable, early apoptotic, and late apoptotic U251 cells after 24 h of treatment with XLB and RA. Compared with those in the CK group, the percentages of viable cells in the XLB40 and XLB80 groups decreased by 9% and 27.13%, respectively; the percentages of early apoptotic cells increased by 3.4% and 7.49%, respectively; and the percentages of late apoptotic cells increased by 5.58% and 17.13%, respectively. In the RA40 and RA80 groups, the percentages of viable cells decreased by 7.53% and 17.43%, respectively; the percentages of early apoptotic cells increased by 3.34% and 8.43%, respectively; and the percentages of late apoptotic cells increased by 4.02% and 8.67%, respectively (Figure 5F). These results indicate that these CPEOs promote apoptosis in U251 cells in a concentration-dependent manner.

### 3.6. Effects of the CPEOs on Ca^2+^ Levels in U251 Cells

**Figure 6:**
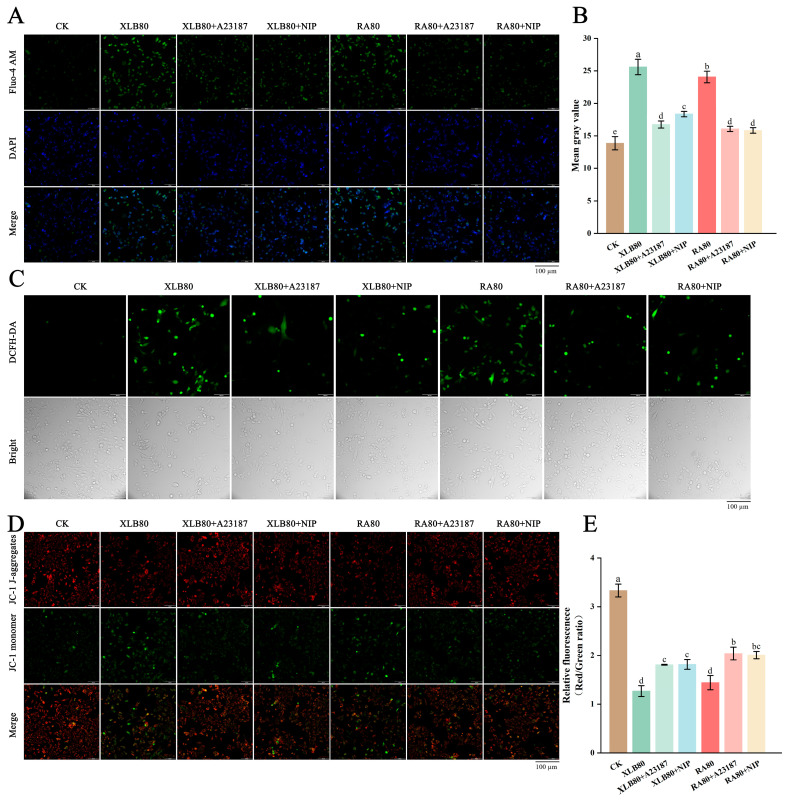
Effects of the CPEOs on Ca^2+^ levels, ROS generation, and the MMP in U251 cells. (**A**) The cell nuclei and intracellular Ca^2+^ in U251 cells after CPEOs treatment were stained with DAPI (blue) and Fluo-4 AM (green), respectively. (**B**) Average fluorescence intensity of Ca^2+^ in different treatment groups. (**C**) Using DCFH-DA (green) to stain ROS in U251 cells treated with CPEOs. (**D**) Fluorescence images of U251 incubated with JC-1 after treatment with XLB and RA EOs. (**E**) MMP quantified by measuring green fluorescence intensity and red fluorescence intensity. XLB80 = 80 µg/mL, RA80 = 80 µg/mL, XLB80 + A23187, XLB80 + NIP, RA80 + A23187, and XLB80 + NIP represents pretreatment with 10 µM of A23187 and NIP for 1.5 h before adding XLB and RA EOs. The data are presented as mean ± SD. Values followed by different superscripts (a–e) are significantly different (*p* < 0.05).

The green fluorescent probe Fluo-4 AM was applied to detect the Ca^2+^ levels in the U251 cells. As shown in Figure 6A, compared with that in the CK group, the green fluorescence intensity significantly increased in the U251 cells in the XLB80 and RA80 groups, whereas it decreased markedly after the addition of the inhibitors. Figure 6B shows that the CPEO treatment increased the intracellular Ca^2+^ content in the U251 cells, with the average fluorescence intensity increasing from 13.86 ± 1.02 in the CK group to 25.60 ± 1.19 and 24.07 ± 0.90 in the XLB80 and RA80 groups, respectively. After the addition of the calcicoptosis inhibitors A23187 and NIF, the intracellular Ca^2+^ content decreased, with the average fluorescence intensity decreasing to 16.74 ± 0.54, 18.35 ± 0.42, 16.06 ± 0.41, and 15.83 ± 0.43, respectively. These results suggest that these CPEOs increase the intracellular Ca^2+^ content in U251 cells, inducing calcicoptosis.

### 3.7. Effects of the CPEOs on ROS Generation and the MMP in U251 Cells

We next evaluated the ROS levels in CPEO-treated U251 cells. As shown in Figure 6C, the XLB80 and RA80 groups presented substantial green fluorescence, indicating that these CPEOs induced ROS accumulation in the U251 cells. However, after the addition of the calcicoptosis inhibitors, the intensity of the green fluorescence within the cells decreased significantly, indicating that the calcicoptosis inhibitors effectively reduced the ROS generation within the cells.

The accumulation of large amounts of ROS is often accompanied by a loss in MMP. A decrease in the red/green fluorescence intensity ratio in cells stained with the dye JC-1 indicates mitochondrial depolarization, which leads to cell death. Healthy U251 cells exhibited red fluorescence, whereas the cells in the XLB80 and RA80 groups presented an increase in green fluorescence intensity and a decrease in red fluorescence intensity, indicating a significant loss of MMP. However, the addition of A23187 or NIF effectively rescued the loss of MMP (Figure 6D). As shown in Figure 6E, compared with those in the CK group, the MMPs in the XLB80 and RA80 groups decreased to 1.27 ± 0.11 and 1.44 ± 0.15, respectively. However, after the pretreatment with the inhibitors, these values increased to 1.81 ± 0.01, 1.82 ± 0.10, 2.04 ± 0.13, and 2.01 ± 0.08, respectively. Therefore, these CPEOs promote the elevation of ROS levels in U251 cells, leading to a loss of MMP.

### 3.8. Effects of the CPEOs on the MDA and GSH Contents in U251 Cells

**Figure 7:**
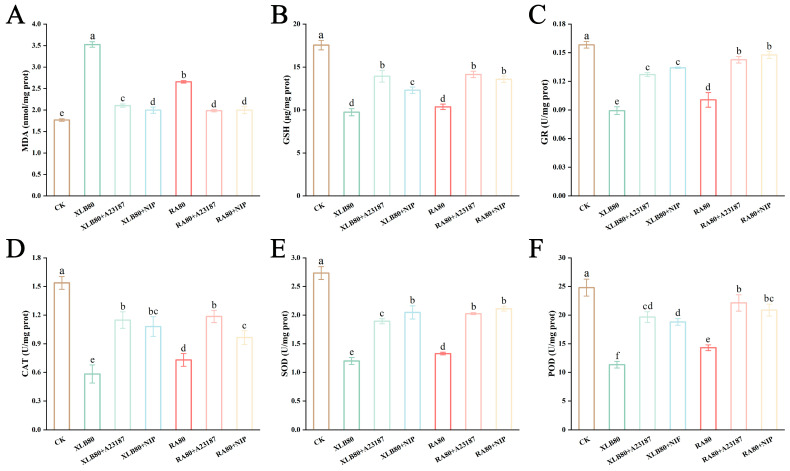
Effects of the CPEOs on MDA (**A**); GSH levels (**B**); and GR (**C**), POD (**D**), SOD (**E**), and CAT (**F**) activities in U251 cells. XLB80 = 80 µg/mL, RA80 = 80 µg/mL, XLB80 + A23187, XLB80 + NIP, RA80 + A23187, and XLB80 + NIP represents pretreatment with 10 µM of A23187 and NIP for 1.5 h before adding XLB and RA EOs. The data are presented as mean ± SD. Values followed by different superscripts (a–f) are significantly different (*p* < 0.05).

Figure 7A shows that, compared with that in the CK group, the MDA contents in the XLB80 and RA80 groups increased by 103.31% and 49.82%, respectively. In the experimental groups in which the calcicoptosis inhibitors were added, the MDA contents decreased by 41.51%, 41.60%, 23.26%, and 23.25% compared with those in the corresponding CPEO-treated groups. Therefore, the treatment with the CPEOs significantly increased the MDA levels in the U251 cells, leading to membrane lipid peroxidation.

Figure 7B shows that, compared with those in CK cells, the GSH levels in the U251 cells treated with XLB80 and RA80 decreased by 40.98% and 36.80%, respectively. However, in the calcicoptosis inhibitor groups, the GSH levels significantly increased compared with those in the corresponding CPEO-treated groups, increasing by 34.65%, 21.10%, 34.34%, and 30.13%, respectively. These results indicate that the EOs XLB and RA can reduce GSH levels, leading to an increase in ROS in U251 cells, and thereby intensifying oxidative stress.

### 3.9. Effects of the CPEOs on the Activities of Antioxidant Enzymes in U251 Cells

As shown in Figure 7C, compared with that in the CK group, the GR activity in the U251 cells in the XLB80 and RA80 groups decreased by 43.58% and 36.44%, respectively. Similarly, after the pretreatment with the calcicoptosis inhibitors, the GR activity in the corresponding groups increased by 42.41%, 50.35%, 41.75%, and 46.75%, respectively. Thus, the CPEOs significantly reduced the GR activity in the U251 cells.

The CAT activities after the XLB80 and RA80 treatment are presented in Figure 7D. The CPEO treatment significantly reduced the CAT activity in the U251 cells by 62.09% and 52.50%, respectively. After the pretreatment with the calcicoptosis inhibitors, the CAT activity in the corresponding groups increased by 97.01%, 85.32%, 62.31%, and 32.03%, respectively. In summary, after 24 h of treatment with the EOs XLB and RA, the CAT activity in the U251 cells was significantly reduced.

The CPEOs significantly inhibited the SOD activity in the U251 cells (Figure 7E). Compared with that in the CK group, the SOD activity in the XLB80 and RA80 groups decreased by 56.18% and 51.42%, respectively. After the pretreatment with the calcicoptosis inhibitors, the SOD activity in the corresponding groups increased by 57.99%, 70.84%, 52.49%, and 59.00%, respectively. Thus, the EOs XLB and RA significantly inhibited the SOD activity in the U251 cells.

As shown in Figure 7F, compared with that in the CK group, the POD activity in the XLB80 and RA80 groups was significantly lower. After the treatment with XLB and RA, the POD activity in the U251 cells decreased by 54.24% and 42.29%, respectively. After the pretreatment with the calcicoptosis inhibitors, the POD activity in the corresponding groups increased by 73.27%, 65.81%, 54.65%, and 45.90%, respectively. These results indicate that the EOs XLB and RA can inhibit POD activity in U251 cells.

### 3.10. Effects of the CPEOs on the Relative Expression of Calcicoptosis-Related Genes in U251 Cells

**Figure 8:**
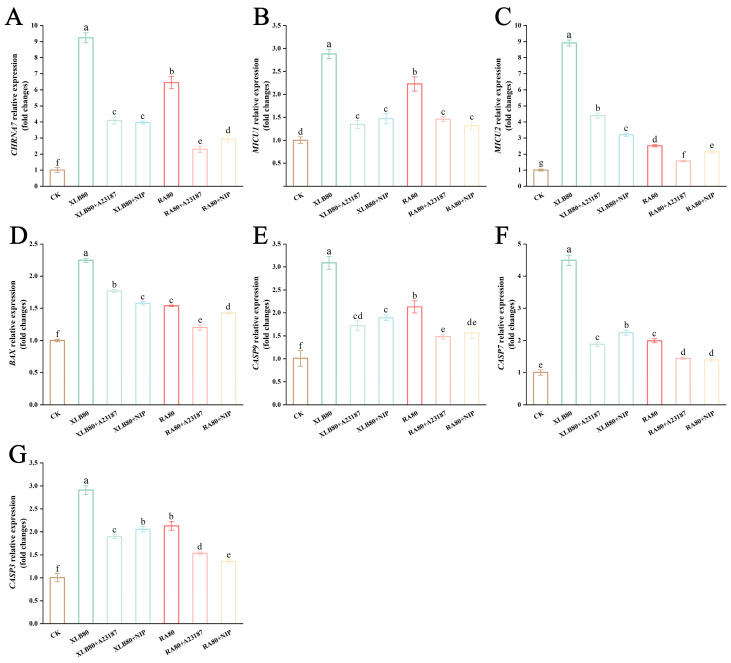
Effect of the CPEOs on the expression of the *CHRNA7* (**A**), *MICU1* (**B**), *MICU2* (**C**), *BAX* (**D**), *Caspase-9* (**E**), *Caspase-7* (**F**), and *Caspase-3* (**G**) in U251 cells. XLB80 = 80 µg/mL, RA80 = 80 µg/mL, XLB80 + A23187, XLB80 + NIP, RA80 + A23187, and XLB80 + NIP represents pretreatment with 10 µM of A23187 and NIP for 1.5 h before adding XLB and RA EOs. The data are presented as mean ± SD. Values followed by different superscripts (a–g) are significantly different (*p* < 0.05).

To further evaluate the effects of the CPEOs on U251 cells, qRT–PCR was used to detect the expression of calcicoptosis-related genes (Figure 8). The expression levels of the Ca^2+^ ion channels cholinergic receptor nicotinic alpha 7 subunit (*CHRNA7*), mitochondrial calcium uptake 1 (*MICU1*) and mitochondrial calcium uptake 2 (*MICU2*), which are located on the cell membrane and mitochondrial membrane, were significantly increased. Compared with that in the control group, the expression of *CHRNA7* mRNA in the XLB80 and RA80 groups increased significantly by 9.17-fold and 6.40-fold, respectively. After the pretreatment with the calcicoptosis inhibitors, the relative expression of *CHRNA7* in the corresponding groups decreased 0.44-fold, 0.43-fold, 0.36-fold, and 0.46-fold, respectively (Figure 8A). Compared with that in the CK group, the expression levels of *MICU1* in the XLB80 and RA80 groups increased 2.88-fold and 2.22-fold, respectively. After the pretreatment with the inhibitors, the expression of *MICU1* in the corresponding groups decreased 0.4-fold, 0.51-fold, 0.65-fold, and 0.59-fold, respectively (Figure 8B). Compared with those in the CK group, the expression levels of *MICU2* in the XLB80 and RA80 groups increased 8.91-fold and 2.5-fold, respectively. However, after the pretreatment with the inhibitors, the *MICU2* expression decreased 0.49-fold, 0.36-fold, 0.62-fold, and 0.85-fold, respectively (Figure 8C).

The results revealed that the expression levels of the proapoptotic gene BCL2 associated X protein (*BAX*) in the U251 cells treated with XLB80 and RA80 significantly changed (Figure 8D). Compared with that in the control group, the expression of the *BAX* gene was significantly upregulated 2.24-fold and 1.54-fold in the XLB80 and RA80 groups, respectively. Additionally, we observed that after the pretreatment with the calcicoptosis inhibitors, the relative expression of *BAX* in the corresponding groups decreased 0.79-fold, 0.70-fold, 0.78-fold, and 0.92-fold, respectively. A significant increase in *BAX* expression indicates the induction of apoptosis via caspase cascade activation, such as *Caspase-3*, *Caspase-7*, and *Caspase-9*. As shown in Figure 8E, compared with that in the control group, the expression of the *Caspase-9* gene in the XLB80 and RA80 groups increased 3.06-fold and 2.11-fold, respectively. After the treatment with the calcicoptosis inhibitors, the relative expression of *Caspase-9* in the corresponding groups decreased 0.56-fold, 0.61-fold, 0.70-fold, and 0.73-fold, respectively. In the XLB80 and RA80 groups, the expression of *Caspase-3* increased 2.90-fold and 2.12-fold, respectively. After the pretreatment with the calcicoptosis inhibitors, the expression of the *Caspase-3* gene in the corresponding groups was significantly downregulated 0.65-fold, 0.71-fold, and 0.72-fold, respectively (Figure 8F). Similarly, in the XLB80 and RA80 groups, the expression of *Caspase-7* increased 4.48-fold and 1.99-fold, respectively. After the pretreatment with the calcicoptosis inhibitors, the expression of *Caspase-7* in the corresponding groups decreased 0.42-fold, 0.50-fold, 0.72-fold, and 0.69-fold, respectively (Figure 8G).

## 4. Discussion

Currently, the standard treatment for GBM involves surgery combined with radiotherapy and chemotherapy (Stupp protocol). Despite achieving some success, the five-year survival rate for GBM patients remains extremely low. The presence of the BBB poses a significant challenge, as many traditional drugs that have proven effective against other tumors have no significant therapeutic effects in GBM treatment [2,3]. Therefore, with the ever-growing interest in exploring natural products as potential cancer treatments, CPEOs have emerged as one of the most valuable botanical products utilized in medical and complementary therapeutic strategies [30]. We had discovered that CPEOs might exert cytotoxic effects on glioblastoma cells by inducing calcicoptosis. This study has offered a new approach for the comprehensive utilization of citrus resources. Furthermore, it has uncovered the mechanism of action of CPEOs as a calcicoptosis inducer, providing a theoretical basis for the development of novel anti-cancer drugs.

In this study, after analyzing the components of five types of CPEOs, it was found that D-limonene had relatively high concentrations in RA and STJ, α-pinene was abundant in XLB and RA, and myrcene was prominent in FJ and RA. D-Limonene is generally recognized as the primary anti-cancer cell proliferation component in CPEOs, capable of inducing mitochondrial damage in proliferating cancer cells, leading to apoptosis [31]. In this study, the five CPEOs killed U251 cells in a concentration-dependent manner. Since RA contained a higher level of D-limonene, it demonstrated stronger inhibitory effects, leading us to speculate that this was the primary factor contributing to RA’s anti-cancer activity. Other research reports have indicated that β-myrcene exhibits strong anti-proliferative effects by inducing DNA damage in cancer cells [32]. Therefore, the significant inhibitory effect of RA on U251 cells can be attributed not only to its high content of d-limonene but also potentially to its relatively high content of β-myrcene. Although XLB was not an essential oil with a high content of D-limonene, its inhibitory effect on U251 cells surpassed that of STJ, which had a higher content of D-limonene. This may have been due to the highest content of α-pinene present in XLB. α-Pinene was an important monoterpene in CPEOs, which could inhibit proliferation by inducing total oxidative stress in N2a neuroblastoma cells [33]. Citrus EOs are complex mixtures of more than 200 compounds [15]. In addition to the major components, the minor components may also contribute significantly to the activity of the EO. For example, citral and 3-carene have been shown to inhibit A549 human lung cancer cells by inducing early apoptosis [34]. In this study, the observed potent inhibitory effects of the CPEOs on U251 cells may also be due to the cumulative effects of both the major and minor components, which enhances tumor cell death. Similarly, Palma et al. [35] reported that the minor components may synergize with D-limonene to inhibit MCF-7 cell proliferation. This finding is consistent with the results of our study, suggesting that these CPEOs may have greater potential for tumor inhibition than limonene or pinene alone.

The disrupted regulation of the cell cycle leads to unrestrained proliferation and migration [18,36] and is an important biological characteristic of malignant glioblastoma [37]. Terpenes can inhibit the further progression and migration of advanced-stage cancer through various cellular and molecular mechanisms, such as blocking the cell cycle and inducing apoptosis [38]. Studies have shown that the EO of *Dysphania botrys* (L.) can arrest HeLa cells in the G0/G1 phase of the cell cycle and promote apoptosis [39]. The D-limonene-rich volatile oil of blood oranges has been found to inhibit the migration of SW480 and HT-29 cells [40]. Some studies have also shown that monoterpenes in CPEOs, such as D-limonene and α-pinene, affect the cell cycle by influencing the expression of cyclin proteins, thereby inhibiting the proliferation of cancer cells [15,41]. After the treatment with the two types of CPEOs in this study, U251 cells were arrested in the G0/G1 phase, possibly due to a similar mechanism affecting the expression of cyclin proteins, which induced cell cycle arrest in response to DNA damage. In this study, both CPEO treatments also arrested U251 cells in the G0/G1 phase of the cell cycle. Research has revealed that the mechanism underlying the ability of rosemary EO to arrest melanoma B16-F10 cells in G1/G0 phase and hinder their proliferation involves the induction of ferroptosis [42]. Metal ion regulation plays a crucial role in the tumor-suppressing effects of EOs. Notably, *Ocimum × africanum* EO can induce endoplasmic reticulum (ER) stress in the human gastric cancer cell line AGS by interfering with protein folding within the ER, leading to the sustained release of endogenous Ca^2+^. This, in turn, triggers apoptosis and reduces cell migration [43]. There are also reports indicating that components of plant EOs can regulate ion channels and receptors [44,45]. For example, D-limonene can activate the TRPA1 channel, triggering an influx of calcium ions [46]. Menthol, a monoterpene, has been shown to activate the ryanodine receptor RyR1, leading to the release of Ca^2+^ that had been stored in the ER/sarcoplasmic reticulum of cells [47]. In the present study, treating U251 cells with CPEOs led to an increase in the concentration of intracellular calcium ions, resulting in severe calcium overload that significantly disrupted normal cellular proliferation. This calcium overload arrested the cells in the G0/G1 phase and reduced their migratory ability. These findings are consistent with those of previous studies, suggesting that citrus fruits may inhibit U251 cells through CPEO-induced calcium overload. Additionally, some EO components can penetrate cell membranes. In another study, eugenol (a monoterpene) was found to modulate sodium and calcium channels in rat dental afferent neurons without requiring TRPV1 activation [48,49]. In this study, the terpenes present in the CPEOs were shown to have a good lipid solubility, which enabled them to freely cross cell membranes. These compounds may directly affect the activities of Ca^2+^ ion channels and further induce ER stress, leading to the release of Ca^2+^.

Intracellular Ca^2+^ overload can damage mitochondria, and damaged mitochondria cause ROS production and excessive ROS accumulation, ultimately leading to cell death [19,22]. ROS-mediated mitochondrial DNA damage can lead to respiratory chain dysfunction, resulting in increased levels of free radicals within the cell [50,51]. Research has shown that bergamot EO can increase ROS production by approximately 80% in neuroblastoma cells, which is toxic to these tumor cells [52,53]. Another study on the EO of Schisandrae semen (Turcz.) Baillon noted that this EO significantly enhanced apoptosis by triggering ROS-mediated mitochondrial dysfunction [54]. In this study, the CPEOs induced Ca^2+^ overload in U251 cells and the mitochondria were damaged, which further led to the excessive accumulation of ROS within these cells. Ca^2+^ overload may be a key mechanism by which CPEOs exert their antitumor effects. Oxidative stress in tumor cells further triggers a large influx of Ca^2+^ into the cell, leading to intracellular calcification and ultimately apoptosis. The ROS level is regulated by antioxidant enzyme systems (such as SOD, POD, CAT, and GR) and nonenzymatic defense systems (such as GSH). These mechanisms eliminate excess intracellular ROS, modulate oxidative stress-induced cellular damage, and maintain normal cellular functions [20]. However, upregulation of the antioxidant defense system in some tumor cells counteracts the ROS accumulation, promoting tumor cell survival and proliferation [55,56]. Resveratrol can inhibit the protective enzyme system in rapidly proliferating cancer cells, causing an imbalance in the expression levels and activities of *SOD*, *CAT*, and *GPX* in tumor cells. This leads to the continuous accumulation of ROS in the mitochondria, thereby inhibiting the cancer cells [10]. In this study, after treatment with the CPEOs, the activities of the intracellular antioxidant enzymes SOD, POD, CAT, and GR were inhibited. *Artemisia argyi* H.Lév. & Vaniot EO and its key active components have been shown to reduce the GSH/GSSG ratio and increase the MDA content, leading to lipid peroxidation in pancreatic cancer cells [57]. In the present study, after treatment with the CPEOs, the activity of intracellular GR was inhibited, resulting in a decreased rate of GSSG reduction to GSH and a significant decrease in the GSH content. Additionally, the level of MDA, a marker of oxidative stress, increased significantly. Therefore, the inhibitory effect of CPEOs on cancer cells is most likely achieved through calcium overload leading to mitochondrial dysfunction, which causes an imbalance in the cellular redox system and ultimately results in oxidative stress.

Intracellular calcium ion homeostasis is controlled by multiple genes. *CHRNA7* is a ligand-gated ion channel that triggers calcium ion flux upon stimulation by choline and acetylcholine [58]. Increased expression of *CHRNA7* may increase the entry of Ca^2+^ into cells [59]. In our study, treatment with the CPEOs significantly upregulated *CHRNA7*, which activated the calcium ion channels on the cell membrane and allowed the influx of many extracellular calcium ions, leading to intracellular Ca^2+^ overload. This may be the initial step in the CPEO-induced apoptosis of U251 cells. Intracellular Ca^2+^ temporarily enters the mitochondria through the mitochondrial calcium uniporter (MCU) located on the mitochondrial inner membrane. *MICU1* and *MICU2* regulate MCU activity by sensing changes in the cytosolic Ca^2+^ content to open and close MCU as needed, thereby preventing the excessive influx of Ca^2+^ [60,61]. A study has shown that l-Borneol, a type of monoterpene, can affect intracellular homeostasis by regulating the relative expression level and protein level of MCU in mitochondria [62]. Under CPEO stimulation, *MICU1* and *MICU2* are significantly upregulated, activating MCU and causing mitochondrial calcium dyshomeostasis, which leads to mitochondrial damage. Many chemotherapeutic drugs exert their anticancer effects by regulating the permeability of the outer mitochondrial membrane to modulate apoptosis [63]. Apoptosis can be mediated by either the mitochondrial pathway or the death receptor pathway. Members of the *BCL-2* family, such as *BCL-2* and *BAX*, are key mediators of mitochondrial pathway-mediated apoptosis [64]. In our study, the treatment with the CPEOs significantly upregulated *BAX* in U251 cells, promoting a change in mitochondrial membrane permeability and triggering apoptosis. The mechanisms by which D-limonene and α-pinene, the key components of CPEOs, upregulate *BAX* and induce apoptosis in cancer cells have been confirmed in T-cell tumor, AGS, HepG2, and MCF-7 cells [65,66,67]. The Caspase family, including *Caspases-3*, *7*, and *9*, is closely associated with the apoptotic process, with *Caspase-3* being considered the “executioner” of apoptosis [68]. D-Limonene and α-Pinene can activate the expression of *Caspase-9* and *Caspase-3*, stimulating apoptosis in cancer cells [31,41]. Our qRT–PCR results indicate that after the disruption of the mitochondrial membrane permeability by the CPEOs, the apoptotic initiator *Caspase-9* is activated, which further targets and activates the apoptotic effectors *Caspase-3* and *Caspase-7* [69]. The findings here suggest that these CPEOs can induce apoptosis in U251 cells through the mitochondrial pathway.

In this study, we explored the application potential of CPEOs, a class of natural products with unique chemical properties, in healthcare and pharmaceuticals. Our research revealed that CPEOs, as compounds with potential antitumor activity, exhibit broad prospects for application in inhibiting tumor cell migration and inducing calcium-dependent cell death. In the future, further research should delve deeper into their underlying molecular mechanisms, particularly the pathways associated with calcicoptosis, and conduct in vivo experiments to fully explore and validate the immense possibilities of CPEOs in promoting human health.

## 5. Conclusions

In this study, using GC–MS, we identified the chemical compositions of five CPEOs, and the antiproliferative activities of CPEOs from five different citrus varieties on U251 cells were screened. Subsequently, the CPEOs XLB and RA, which exhibited better antiproliferative effects, were selected for further exploration of their mechanisms against glioblastoma cells. Our study revealed that CPEOs can promote intracellular and mitochondrial calcium ion dysregulation through *CHRNA*, *MICU1*, and *MICU2*, leading to mitochondrial dysfunction. This resulted in increased ROS levels, a decrease in the MMP, and elevated lipid peroxidation, triggering calcicoptosis and inhibiting the proliferation of U251 cells by arresting them in the G1/G0 phase. This research highlights the potential anticancer mechanisms of CPEOs and provides a theoretical reference for subsequent resource utilization and development. Further molecular experiments will be conducted to elucidate the mechanisms by which CPEOs act against glioblastoma.

## Figures and Tables

**Table 1:** Information on citrus materials.

No.	Citrus Resources	Latin Name	Locality (China)	Abbreviation
1	XingLuBiXiYou	*Citrus paradisi* Macf.	Zhangzhou, Fujian	XLB
2	RongAnJinDan	*Fortunella crassifolia* Swingle cv. Chintan	Rongan, Guangxi	RA
3	FengJieQiCheng	*Citrus sinensis* Osbeck cv. Fengjieqicheng	Fengjie, Chongqing	FJ
4	JinCheng26	*Citrus sinensis* Osbeck cv. Jincheng26	Jiangjin, Chongqing	JC26
5	ShaTangJu	*Citrus reticulata* ‘Shatang’	Guilin, Guangxi	STJ

## Data Availability

All of the data are contained within the article and the Supplementary Materials.

## Supplementary Materials

The following supporting information can be downloaded at: https://www.mdpi.com/article/10.3390/antiox14010011/s1, Figure S1: The calcicoptosis inhibitors enhances the cell viability treated by CPEOs; Figure S2: The Total Ion Chromatogram (TIC) of essential oils extracted from the peels of five citrus varieties; Table S1: Primers used for real time-quantitative polymerase chain reaction (RT-qPCR) of calcicoptosis-related genes in U251 cells; Table S2: Composition of the essential oils extracted from the peels of five citrus fruits.
