# Supplementary material for: Essential Oils from Citrus Peels Promote Calcium Overload-Induced Calcicoptosis in U251 Cells
Source: Antioxidants (Basel). 2024 Dec 25;14(1):11. doi: 10.3390/antiox14010011 (PMC11762846; doi:10.3390/antiox14010011)
Supplement: Supplementary file 1 [file antioxidants-14-00011-s001.zip › antioxidants-3378505-supplementary.pdf]

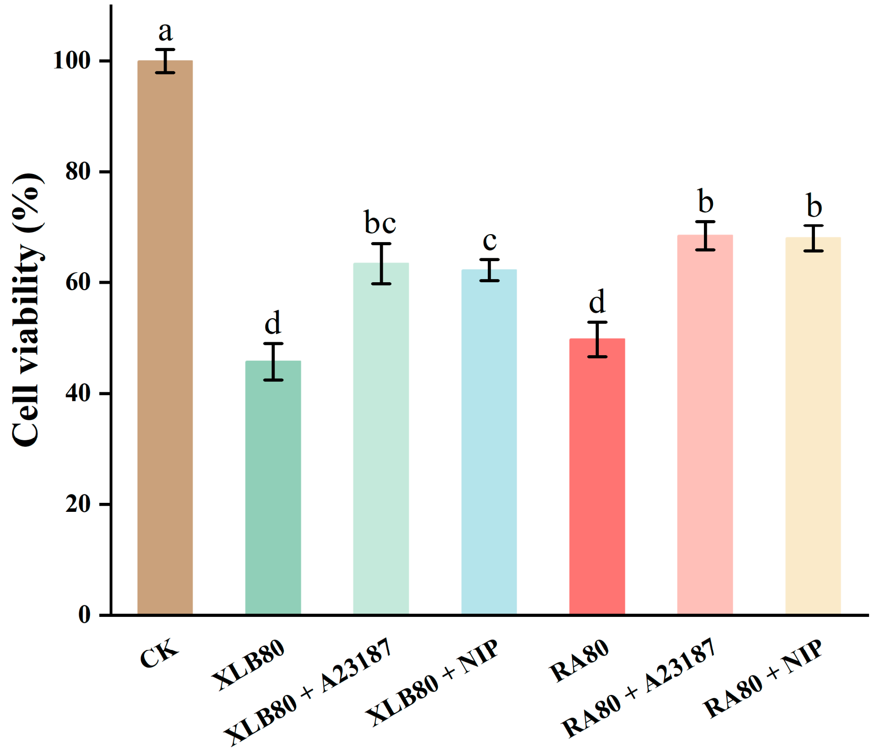

Figure S1. The calcicoptosis inhibitors enhances the cell viability treated by CPEOs. XLB80 = 80 μg/mL, RA80 = 80 μg/ mL, XLB80 + A23187, XLB80 + NIP, RA80 + A23187, and XLB80 + NIP represents pretreatment with 10μM of A23187 and NIP for 1.5 hours before adding XLB and RA EOs. The data are presented as mean ± SD. Values followed by different superscripts (a–d) are significantly different ($p < 0.05$).

A

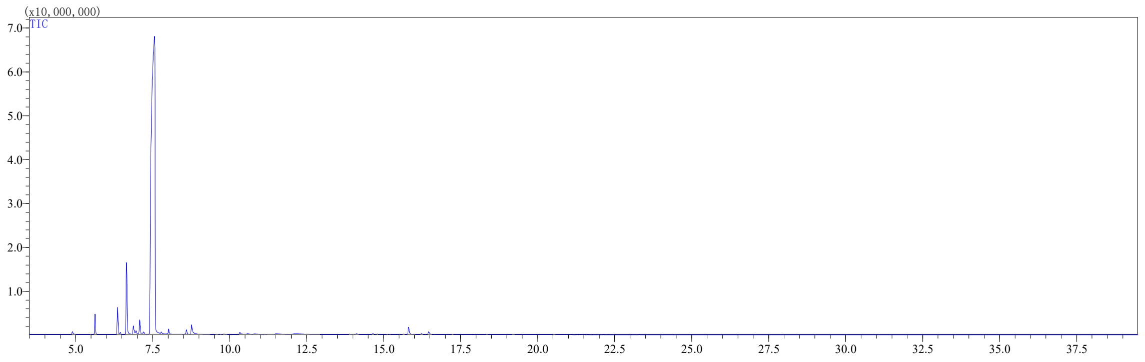

B

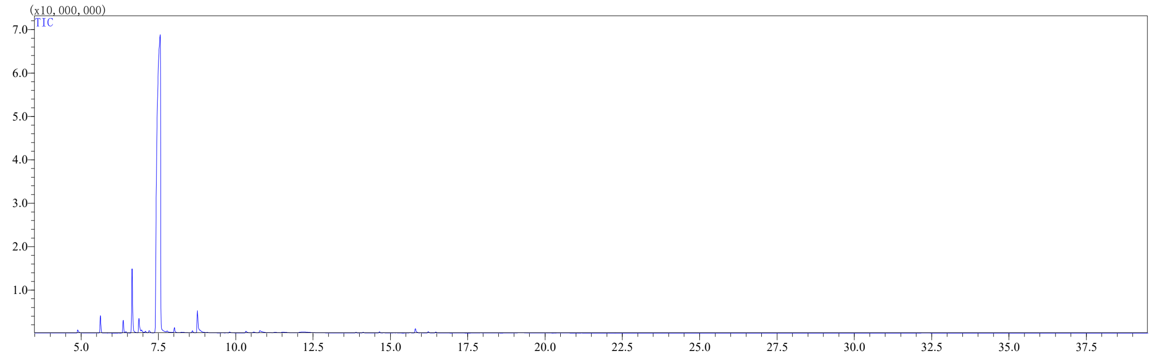

C

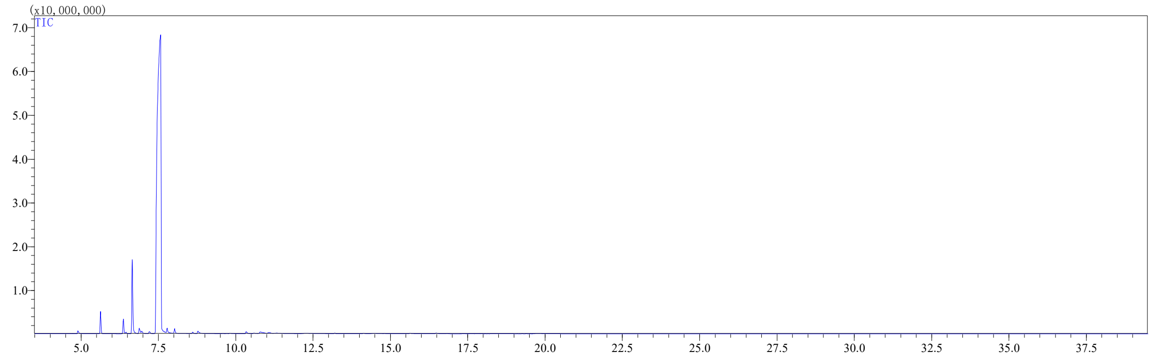

D

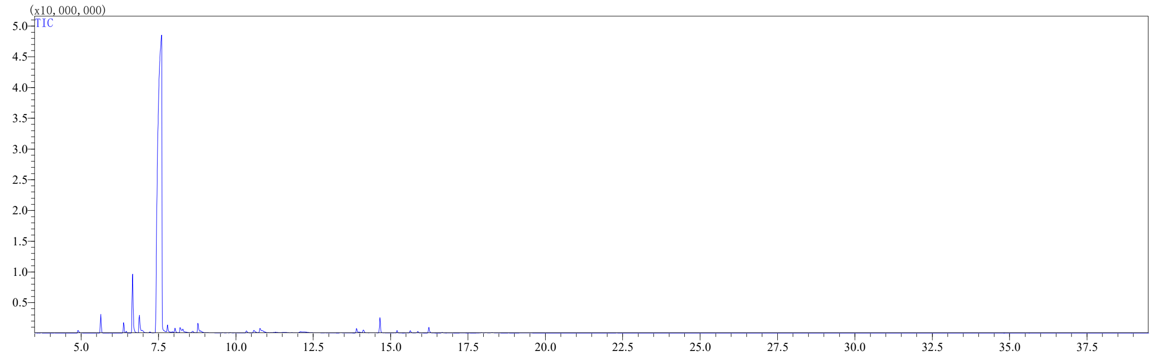

E

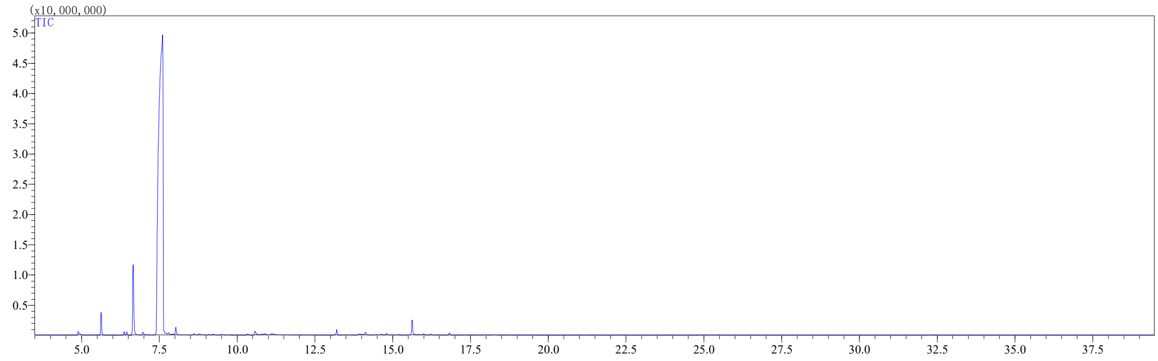

Figure S2. The Total Ion Chromatogram (TIC) of essential oils extracted from the peels of five citrus varieties. FJ (A), JC26 (B), STJ (C), XLB (D), and RA (E). The abscissa represents the time, while the ordinate represents the abundance.

Table S1. Primers used for real time-quantitative polymerase chain reaction (RT-qPCR) of calcicoptosis-related genes in U251 cells.

Target Gene	Gene accession number	Primer Sequences (5′–3′) Forward (Fw) and Reverse (Rw)	Length (bp)	Annealing Temperature (°C)	Fragment Size (bp)
GAPDH	NM_001357943	F: GACCTGCCGTCTAGAAAAAC<br>R: TTGAAGTCAGAGGAGACCAC	20<br>20	56	126
Bax	NM_138763	F: GGCTATTTCAACCAGGGTTCC<br>R: TGCGAATCACCAATGCTGT	21<br>21	60	155
CASP-9	NM_032996	F: CTCAGACCAGAGATTCGCAAAC<br>R: GCATTTCCCCTCAAACTCTCAA	22<br>22	60	116
CASP-7	NM_004346	F: CGGTCCTCGTTTGTACCGTC<br>R: CGCCCATACCTGTCACTTTATCA	20<br>23	60	175
CASP-3	NM_004346	F: CATGGAAGCGAATCAATGGACT<br>R: CTGTACCAGACCGAGATGTCA	22<br>21	60	139
CHRNA7	NM_000746	F: GCTGGTCAAGAACTACAATCCC<br>R: CTCATCCACGTCCATGATCTG	22<br>21	60	106
MICU1	NM_001195519	F: AGAGCATCATTCGCTCCCAAA<br>R: GCAGTTTACGCTGAAATTCGAG	21<br>22	60	171
MICU2	NM_152726	F: AAACTTCAGTCAAGAAGCTGACA<br>R: TAGCCCTTTATCGCCAAGGTC	23<br>21	60	110

Table S2. Composition of the essential oils extracted from the peels of five citrus fruits.

Compound	Retention Index	Retention Time	FJ	JC26	STJ	XLB	RA
			Relative percent content (%)				
**hydrocarbon**							
α-Thujene	902	5.49	0.03	0.02	0.02	0.01	0.01±0.01
α-Pinene	948	5.628	1.39±0.02	1.22±0.01	1.39±0.05	1.68±0.02	1.47±0.01
Camphene	943	5.855	0.01±0.01	0.01	0.01	0.01	0.01
β-Phellandrene	964	6.363	1.05±0.02	0.91	1.01±0.04	0.65±0.01	0.24
β-Pinene	943	6.449	0.19±0.01	0.13±0.01	0.13±0.01	0.13	0.23
β-Myrcene	958	6.651	5.62±0.06	5.15±0.02	5.47±0.08	4.4±0.05	5.56±0.02
α-Phellandrene	969	6.959	0.43±0.01	0.37	0.28±0.05	0.37±0.01	0.24±0.01
α-Terpinene	998	7.223	—	—	—	0.17±0.01	—
D-Limonene	1018	7.563	81.88±0.02	83.24±0.6	84.43±0.65	82.37±0.52	85.24±0.26
β-cis-Ocimene	976	7.781	0.11±0.01	0.07	0.35±0.02	0.39±0.01	0.07
γ-Terpinene	998	8.019	0.34	0.35±0.01	0.36±0.02	0.25±0.01	0.45±0.01
(+)-4-carene	919	7.204	0.69±0.01	0.47	0.38±0.05	0.16	0.17
γ-Elemene	1431	13.209	—	—	0.05	0.01±0.01	0.05±0.01
α-Cubebene	1344	16.396	—	—	0.02±0.01	0.17±0.23	0.05±0.01
α-Copaene	1221	13.898	0.06	0.03±0.01	—	0.45±0.11	0.16±0.05
γ-Muurolene	1435	15.635	—	—	—	0.61±0.03	—
β-Caryophyllene	1494	15.115	0.15±0.04	0.1±0.01	—	0.94±0.09	0.19±0.02

β-Cuvebene	1339	14.79	0.15	0.16	—	0.03	—
α-Guaiene	1490	14.903	—	—	—	0.03±0.01	—
cis,cis,cis-1,1,4,8-Tetramethyl -4,7,10-cycloundecatriene	1579	15.198	—	0.08±0.01	—	0.19±0.01	0.07±0.01
β-Cedrene	1398	15.335	—	—	—	0.03±0.01	—
γ-Cadinene	1435	14.121	—	—	—	—	0.24±0.02
Germacrene B	1603	14.798	—	—	0.05	0.17±0.01	0.41±0.03
δ-Guaiene	1490	15.996	0.08±0.08	—	—	0.03±0.01	—
(-)-β-Cadinene	1440	15.71	0.13±0.01	0.17±0.01	0.05	0.52±0.02	1.3±0.03
(+)-3-carene	948	7.08	1.07±0.02	0.21±0.01	—	—	0.05±0.02
(+)-Cyclosativene	1125	13.753	0.02±0.01	—	—	—	—
(-)-β-Elemene	1398	14.123	0.14	0.07	0.18	—	0.34±0.02
(+)-Sativene	1339	14.262	0.02±0.01	—	—	—	—
α-Caryophyllene	1579	15.2	0.03	—	0.03	—	—
γ-Gurjunene	1461	15.526	0.03	—	—	—	—
(-)-α-Gurjunene	1419	15.994	—	—	—	—	0.12±0.03
(+)-Valencene	1474	15.518	0.02±0.01	0.51±0.01	0.02	—	—
p-Mentha-1,3,8-triene	1029	9.079	—	—	0.03	—	0.07±0.01
δ-EIemene	1377	13.197	—	—	0.09	—	0.43±0.02
(-)-β-Bourbonene	1339	14.054	—	—	—	—	0.05±0.01
α-Ylangene	1221	13.815	—	—	—	—	0.03±0.01
(-)-Aristolene	1403	14.98	—	—	—	—	0.06±0.01
(+)-Aromadendrene	1386	15.097	—	—	—	—	0.06±0.01
(+/-)-epi-β-santalene	1435	15.335	—	—	—	—	0.03±0.01
α-Longipinene	1403	16.378	—	—	—	—	0.12±0.01
Isocaryophyllene	1494	15.115	—	0.05±0.01	0.12±0.02	—	—
Germacrene D	1515	15.638	—	0.04	—	—	—
Ylangene	1221	13.404	—	0.36±0.01	—	—	—
Seychellene	1275	13.754	—	0.42±0.34	—	—	—
**Aldehydes**							
Octanal	1005	6.873	1.14±0.01	1.3±0.01	1.14±0.06	1.45±0.03	—
Nonanal	1104	8.843	—	0.45±0.01	—	0.27±0.01	—
β-Citronellal	1125	9.815	—	—	—	0.06±0.01	—
Decanal	1204	10.804	0.27±0.01	0.21±0.01	0.83±0.07	0.61±0.18	—
β-Citral	1174	11.537	—	0.20±0.09	—	0.14±0.04	—
α-Cyclocitral	1175	9.173	0.01±0.01	—	—	—	—
β-Cyclocitral	1204	11.138	0.04±0.01	0.07±0.02	—	—	—
Neral	1174	11.512	0.55±0.08	—	—	—	—
α-Sinensal	1646	19.638	0.03±0.01	0.05±0.02	0.29±0.03	—	—
p-Menth-1-en-9-al	1175	11.079	—	0.59±0.01	0.30±0.01	—	—
(R)-(+)-Citronellal	1125	9.801	0.09±0.01	0.12	0.12±0.01	—	—

(-)-Perillaldehyde	1207	12.159	—	—	0.42±0.02	—	—
Undecanal	1303	12.586	—	—	0.08±0.03	—	—
Tetradecanal	1601	14.357	—	—	0.29±0.03	—	—
(Z)-7-Hexadecenal	1808	18.441	—	—	0.03	—	—
**Alcohols**							
1-Octanol	1059	8.293	0.83±0.02	0.08±0.05	0.03±0.01	0.45±0.06	—
Linalool	1082	8.764	1.18±0.02	1.09±0.02	0.45±0.03	1.15±0.04	0.17±0.01
p-Mentha-2,8-dien-1-b-ol	1109	9.253	0.02	—	—	0.03	—
1-Nonanol	1159	10.203	—	—	—	0.02±0.01	—
Terpinen-4-ol	1137	10.339	0.32±0.01	0.32	0.33±0.02	0.22±0.01	0.13±0.01
α-Terpineol	1143	10.572	0.24	0.32±0.01	0.18±0.01	0.36±0.01	0.5±0.02
(-)-cis-Carveol	1206	11.113	—	—	0.5±0.03	0.07±0.01	0.39±0.02
Citronellol	1179	11.21	—	—	—	0.22±0.04	—
Elemol	1522	16.66	0.02±0.01	—	0.06±0.01	0.09±0.01	0.03±0.01
Nerolidol	1352	16.904	—	—	—	0.04±0.01	—
γ-Eudesmol	1626	17.93	0.03±0.01	0.04±0.01	0.02	0.06±0.02	0.06±0.01
δ-Cadinol	1580	18.072	—	0.09±0.01	—	0.09±0.01	0.15±0.01
α-Eudesmol	1598	18.27	—	—	—	0.12±0.01	—
Farnesol	1710	19.265	—	0.15±0.05	—	0.08±0.01	—
β-Terpineol	1158	9.693	0.02	0.06±0.01	0.03	—	0.02
(-)-globulol	1530	17.267	—	—	—	—	0.07±0.04
D-Citronellol	1179	11.298	0.2	—	—	—	—
(E)-11(12-Cyclopropyl)dodecen-1-ol	1765	16.463	0.39±0.03	—	—	—	—
trans-p-Mentha-2,8-dienol	1120	9.228	—	—	—	—	0.12±0.01
cis-p-Mentha-2,8-dien-1-ol	1140	9.506	—	—	—	—	0.1
α-Cadinol	1580	18.236	—	—	—	—	0.09±0.01
β-Citronellol	1175	11.286	—	0.04	—	—	—
(Z,E)-tetradeca-9,12-dienol	1672	12.564	—	0.19±0.01	0.16±0.01	—	—
Hedycariol	1694	16.651	—	0.03	—	—	—
β-Nerolidol	1564	16.85	—	0.03±0.01	—	—	—
trans-p-Menth-2-en-1-ol	1109	9.231	—	—	0.02	—	
Tetrahydrolavandulol	1066	12.094	—	—	0.08±0.02	—	—
Juniper camphor	1647	18.358	0.04±0.01				
**Esters**							
Hexyl acetate	984	7.098	—	—	—	0.09±0.01	—
Neryl acetate	1352	9.098	0.06±0.06	—	—	0.04	0.1±0.02
Octyl acetate	1183	10.852	—	—	—	0.42±0.06	0.29±0.02
α-Terpinyl acetate	1333	13.375	—	—	—	0.12±0.16	—
Hexyl butyrate	1183	10.5	0.06	0.24	—	—	—
Geranyl acetate	1352	13.949	—	—	—	0.02	0.15±0.01

p-Mentha-1,8-dien-7-yl acetate	1384	14.471	—	—	—	—	0.14±0.01
Octyl octanoate	1779	19.795	—	0.08±0.02	—	—	—
Citronellol acetate	1302	13.386	—	—	0.08±0.01	—	—
**Ketones**							
Nootkatone	1645	20.53	0.07	0.05	—	—	—
D-Carvone	1190	11.585	—	—	0.12±0.01	—	—
